# Management of Untreated Horizontal Root Fracture: A Case Report

**DOI:** 10.7759/cureus.28133

**Published:** 2022-08-18

**Authors:** Prachi Taori, Pradnya Nikhade, Manoj Chandak, Anuja Ikhar, Joyeeta Mahapatra

**Affiliations:** 1 Department of Conservative Dentistry and Endodontics, Sharad Pawar Dental College, Datta Meghe Institute of Medical Sciences Deemed University, Wardha, IND

**Keywords:** conservative management, fibre post, mta apexification, horizontal root fracture, trauma

## Abstract

Traumatic dental injuries often occur to the teeth and their supporting tissues and they are the main reasons for an emergency visit to a dental clinic. Horizontal root fractures usually are characterized by a fracture line that is perpendicular to the long axis of the root. Root fractures are diagnosed through clinical and radiographic examination. Treatment depends on the position of the fracture, the extent of root involvement, correct diagnosis, clinical management, and radiographic follow-up. This article presents endodontic management of horizontal root fracture using a fibre post. A 28-year-old male patient presented with a horizontal fracture of the maxillary left central incisor at the junction of the apical and middle third of the root. Root canal treatment followed by MTA apexification of the coronal fragment and fibre post gave satisfactory results.

## Introduction

Root fracture can be defined as a “fracture that involves cementum, dentin and pulp” [1]. This type of dental injury is relatively uncommon. Among all the traumatic dental injuries, root fractures account only for 0.5%-7%. Most commonly, horizontal root fractures are seen in the middle third of the root compared to the coronal and apical third [2]. Due to the position of the maxillary central incisor in the dental arch, the chance of occurrence of traumatic dental injuries is more in these teeth (approximately 68%). This is followed by maxillary lateral incisors (27%) and mandibular incisors (5%) [3,4].

The chance of occurrence is more in male patients which may be due to trauma associated with automobile accidents, sports injuries, fights, etc. A root fracture is frequently seen in teeth that are permanent and fully erupted having complete formation of the apex. This may be due to the presence of support through bone and periodontium [1]. 

For accurate diagnosis of root fracture, the proper clinical and radiographic examination should be done like Intraoral periapical radiograph and cone-beam computed tomography. Pulp vitality and mobility of the coronal fragment also should be evaluated. On radiographic examination, a radiolucent line is seen which separates the coronal and the apical fragment. The angular displacement imaging technique is one of several techniques and diagnostic methods to identify the correct angulation of fracture [5].

Factors that are considered for determining the prognosis regarding the vitality of pulp following luxation injury are the factors that are considered in case of healing after horizontal root fracture. Andreasen et al. in their study found that necrosis of pulp after horizontal root fracture occurs in nearly 25% of the cases [6]. Splinting of teeth is done whenever required by repositioning and stabilizing the teeth in their correct position. Following this injury periodic checking of teeth is done to evaluate the vitality of pulp. But evaluation of pulp vitality can be difficult in these cases. Actual pulp vitality can be determined even after several months as said by Feiglin [6,7]. The vitality test actually does not indicate the true status of the pulp as here the nerve supply is damaged & non-functional but the blood supply remains intact.

## Case presentation

**Figure 1:**
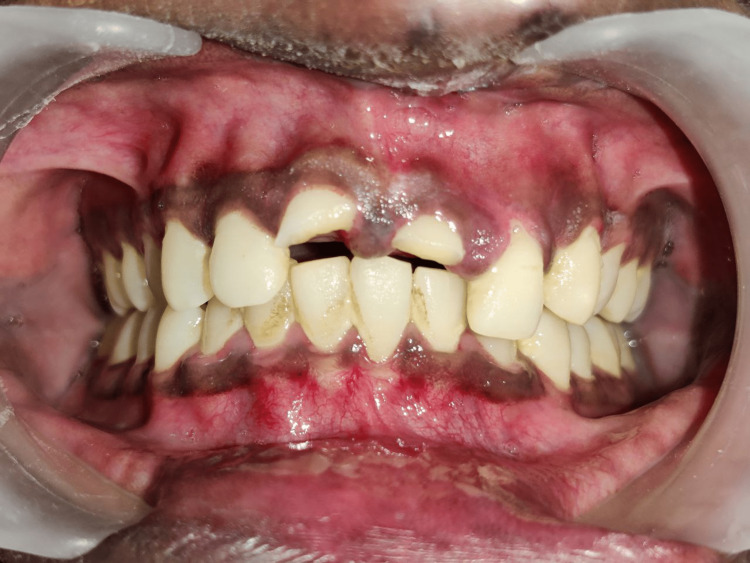
Preoperative clinical photograph (labial view) demonstrating complicated crown fractures of maxillary right and left central incisor

**Figure 2:**
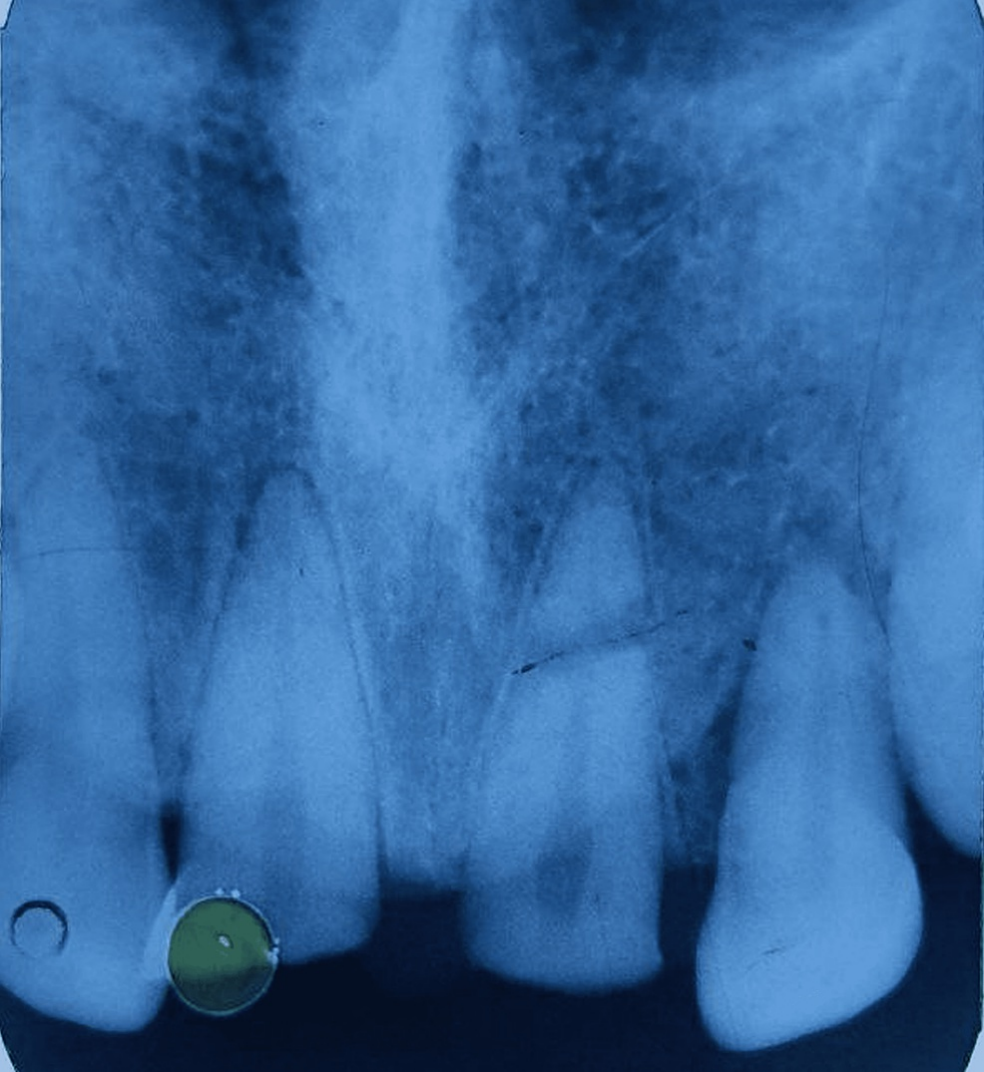
Preoperative radiograph showing horizontal root fracture at the junction of apical and middle third of maxillary left central incisor

A 28-year-old male patient reported to the Department of Conservative Dentistry and Endodontics, Datta Meghe Institute of Medical Science, Wardha, India after trauma in the upper front region because of a road traffic accident nearly 15 days back. The patient complained of fractured teeth in the upper anterior region and desired to replace the same. Past medical history was not significant. On clinical examination, a complicated crown fracture of maxillary right and left central incisor was seen and maxillary left central and lateral incisors were grade II mobile according to Miller's classification of tooth mobility (Figure 1). On radiographic examination, the maxillary left central incisor revealed a horizontal root fracture at the junction of apical and middle third of the root (Figure 2). An intraoral periapical radiograph was taken using bisecting angle technique with the traditional film (size 1).

 

**Figure 3:**
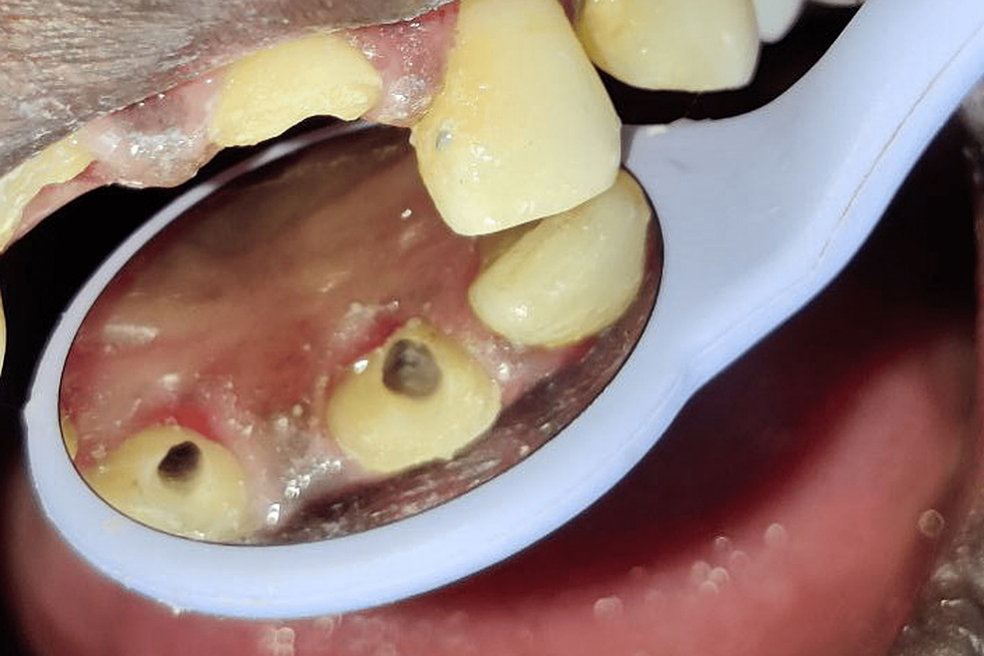
Access opening with maxillary right and left central incisor

**Figure 4:**
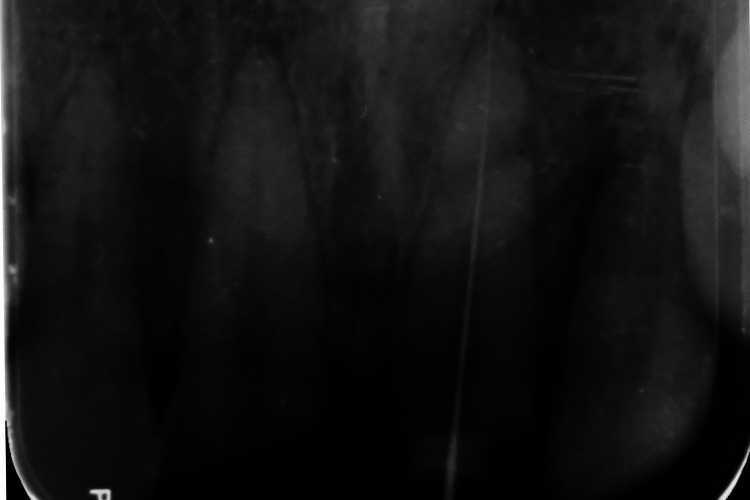
Working length of maxillary left central incisor

The treatment plan was explained to the patient and his consent was obtained. Root canal treatment was initiated in maxillary right and left central incisor. Access opening was done using round bur (BR45 Bur, Mani, Japan) and safe end bur (EX24 Bur, Mani, Japan) (Figure 3). Following pulp extirpation, working length was determined correctly using an electronic apex locater (J Morrita Root Zx Mini) and radiograph. Working length of 21 was found to be 14 mm (Figure 4) and that of 11 was 15 mm. Both the coronal and apical fragments were cleaned and shaped using the K file in a step-back manner with apical file size #60. The canal were irrigated thoroughly using 3% sodium hypochlorite (Parcan, Septodont, India) and 0.9% normal saline for the removal of remaining pulp tissue and debris. This was done using a side vented irrigation needle (Waldent Endodontic Canal Irrigation needle). The triple antibiotic paste was then placed as an intracanal medicament and a temporary pack was given (T-fill, Belgium). The triple antibiotic paste was made using Ciprofloxacin (200mg), Metronidazole (500mg), and Minocycline (100 mg) in the proportion of 1:1:1.

On the second visit, the temporary dressing was removed and the canal was irrigated using saline and an endoactivator (Dentsply, USA) to remove the triple antibiotic paste from the canal. The triple antibiotic paste was placed once again in the canal and a temporary pack was given. Access opening was done with maxillary right and left lateral incisor. After working length determination of these teeth ( 12-18mm and 21-19mm), biomechanical preparation was done using a K file up to apical file size of #60. The triple antibiotic paste was placed in both the teeth and a temporary pack was given.

**Figure 5:**
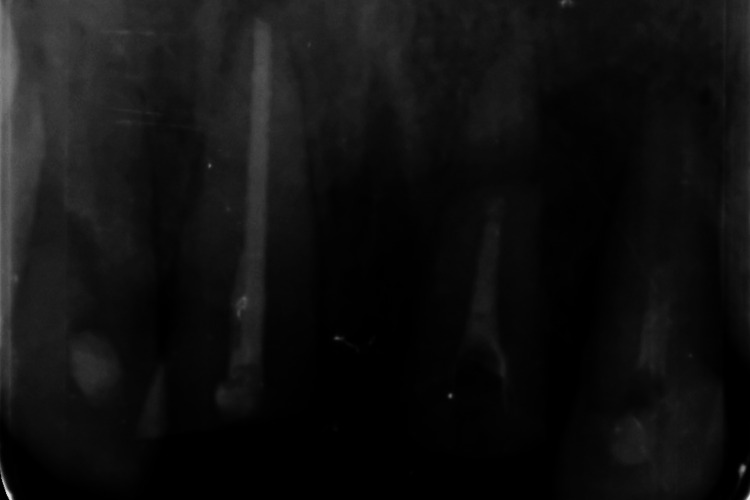
MTA apexification with maxillary left central incisor and obturation with maxillary right central incisor

On the third visit, the temporary dressing was removed and the canal was irrigated using saline and an endoactivator (Dentsply, USA) to remove the triple antibiotic paste from the canal. MTA apexification was done with the coronal fragment of 21. 4mm of the apical plug was placed with 21 and obturation was done with 11 (Figure 5). The triple antibiotic paste was placed in 12 and 22. A temporary pack was given with 11, 12, 21 and 22.

On the fourth visit, the temporary dressing was removed from 12 and 22 and canal was thoroughly irrigated using saline and an endoactivator (Dentsply, USA) to remove the triple antibiotic paste from the canal. Obturation was done with 12 and 22.

**Figure 6:**
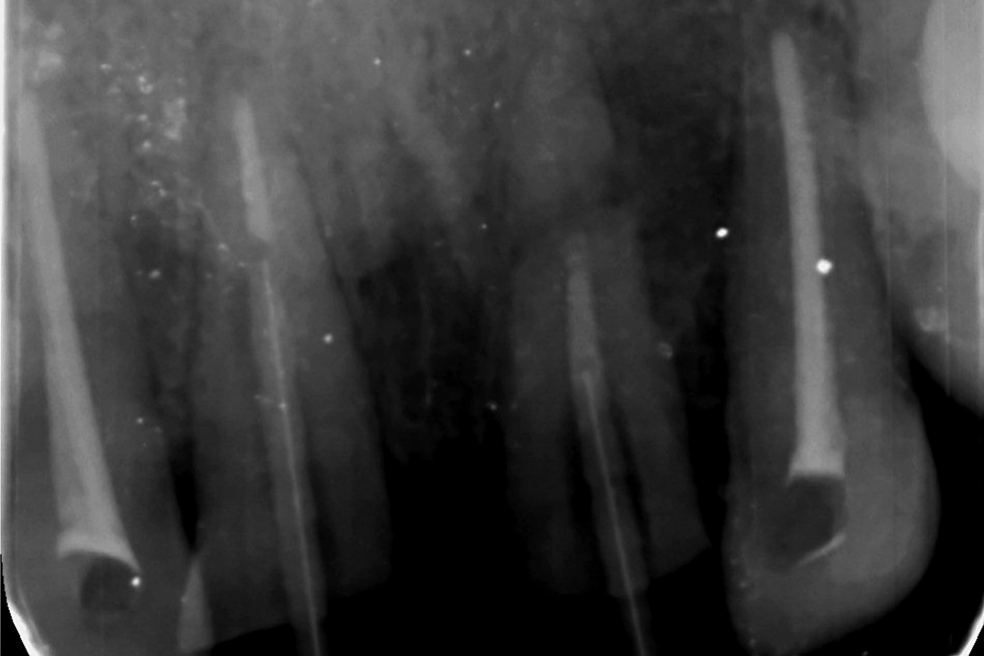
Post fit with maxillary right and left central incisor

**Figure 7:**
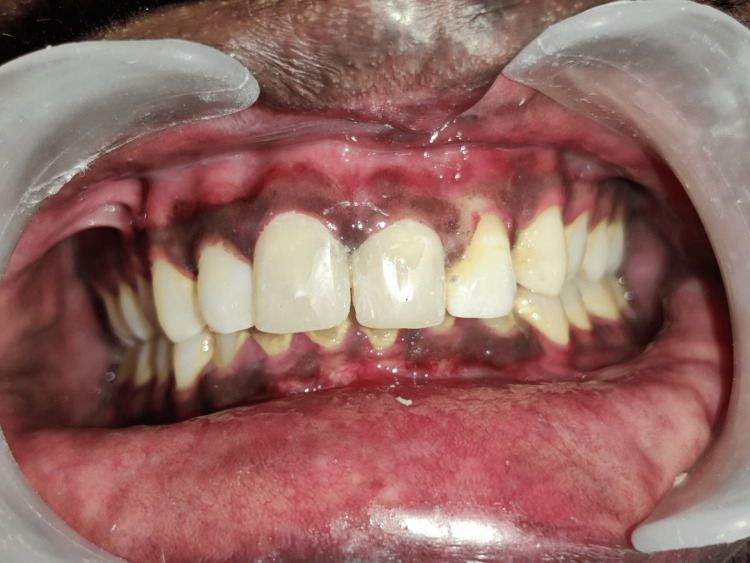
Clinical photograph after post and core treatment of maxillary right and left central incisors (labial view).

**Figure 8:**
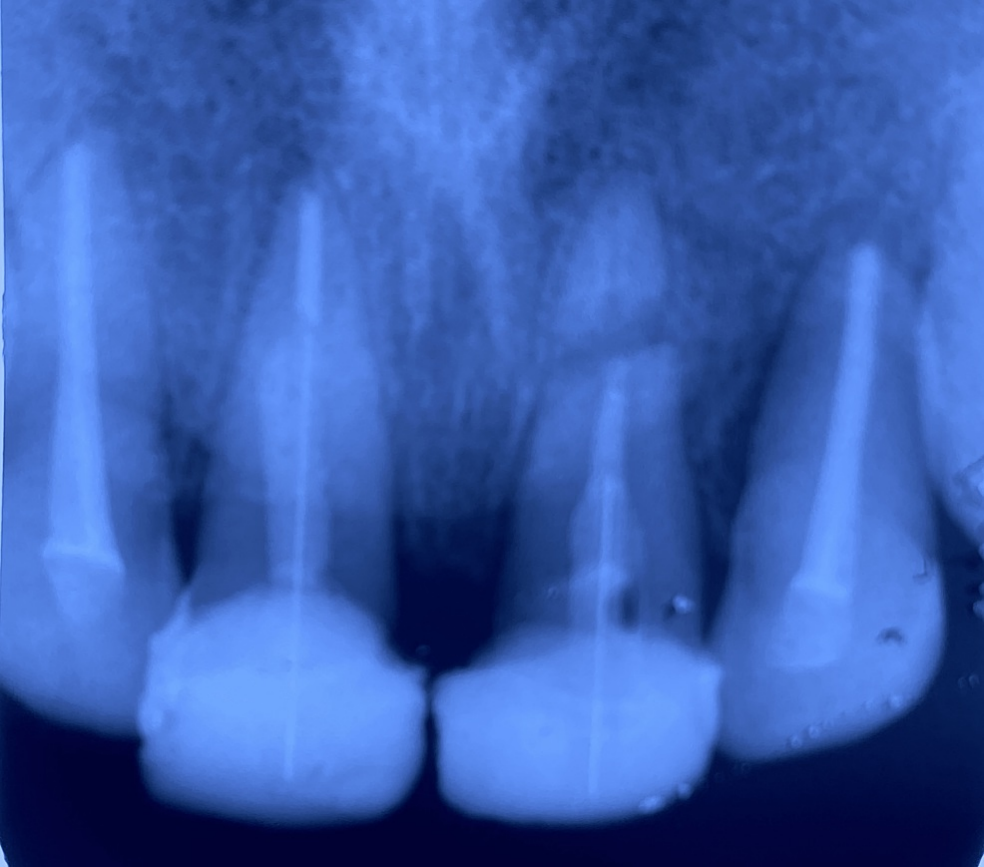
Postoperative radiograph

**Figure 9:**
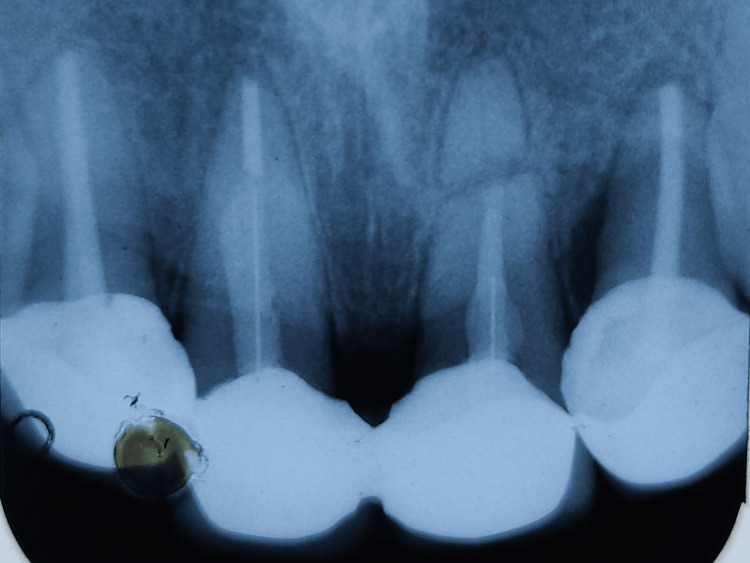
Three months follow-up radiograph

On the fifth visit, post space was prepared up to 9mm from the coronal reference point of 11 and 21 using a Peeso reamer from size 1 to 4. Glass fibre post fit was checked in both the teeth and it was confirmed using a radiograph (Figure 6). Etching of root canals was done using 37% phosphoric acid gel and canals were dried using paper points (Meta paper points). Bonding agent (Dentsply) was applied in canals of both the teeth and was cured for 20 seconds using Woodpecker LED Curing Light unit of intensity 1,200mW/cm^2^. Luting of the post was done using dual cure resin cement (Calibra, Dentsply), inserted into the canal without applying any pressure, and then light cured for 40 seconds. The core build-up was done using composite resin (Figures 7, 8). Post endodontic glass ionomer cement (GC Fuji II) restoration was done with 12 and 22. This was followed by porcelain fused to the metal prosthesis with 12, 11, 21 and 22 (Figure 9).

 

 

## Discussion

Preserving natural dentition and restoring its normal function is one of the goals of dentistry. Extraction of the tooth and its replacement with an osseointegrated implant should always be considered the last treatment option when all other means of retaining the natural tooth have been tried [8].

MTA is a highly recommended material in teeth that has necrotic pulp and an open apex. Various studies have compared the apical closure using calcium hydroxide and MTA. And it has been observed that the success rate as seen clinically and radiographically, it is higher with MTA in terms of fracture resistance, hard tissue formation and inflammation. Therefore, MTA was selected for this case as it might improve the outcome of the treatment [9].

In this case, a fibre post was used. The modulus of elasticity of fibre post is similar to that of dentin, which minimizes the chances of root fracture. According to Gurtu and Singhal [10], using post provides support and stability to the tooth. As the restoration is subjected to tangential stress, the post helps to retain the fractured fragment and also provides strength to the restoration complex. It also creates a monoblock between the post, reconstructive material, cement and the tooth. Also, it prevents dislodgement due to due to nonaxial forces. Fibre post is preferred as it provides good aesthetics and is able to restore the function of a such compromised tooth.

As the cement has higher viscosity and pressure is not applied, this helps in reducing the flow of resin. The amount of resin used should be limited such that a desirable bond is achieved between the post and the dentin. Glass fibre posts were selected as their modulus of elasticity is similar to that of dentin and also they have an added advantage of higher fatigue and tensile strength. When we consider the prognosis of permanent teeth with root fracture, this is related to the amount of dislocation, stage of root development at the time of injury and to some extent, it also depends upon whether the treatment was done.

Andreasen et al. [6] proposed the sequelae to root fracture which can be divided into the following four types (Table 1) [1]. In such cases, long-term follow-ups are required to check if there is any pathological alteration.

**Table 1:** Sequelae to root fracture

Sr no.	Sequela to Root fracture
1]	Healing with calcified tissue – there is close contact between fragments but the fracture line can be seen radiographically.
2]	Healing with interproximal connective tissue – when seen radiographically, the separation between the fragments is seen as narrow radiolucent line and the fractures edges appear narrow.
3]	Healing with interproximal bone and connective tissue – the separation between fractured fragment as seen radiographically is through distinct bony bridge.
4]	Interproximal inflammatory tissue without healing – fracture line seems to widen when seen on radiograph.

## Conclusions

“Preservation of natural dentition and restoration of the oral cavity to a normal functional state” is the primary goal in dentistry. Maintaining the position of fractured teeth in the dental arch is the main objective of treating such teeth. In recent times there has been tremendous improvement in bonding agents and restorative resin and also various new materials are available including the fibre post and dual-cure resin cement that has provided clinicians with different treatment options for the management of fractured roots.

## References

[1] Andreasen FM, Andreasen JO (2007). Crown fractures. Textbook and Color Atlas of Traumatic Injuries to the Teeth.

[2] Bramante CM, Menezes R, Moraes IG, Bernardinelli N, Garcia RB, Letra A (2006). Use of MTA and intracanal post reinforcement in a horizontally fractured tooth: a case report. Dent Traumatol.

[3] Andrade ES, de Campos Sobrinho AL, Andrade MG, Matos JL (2008). Root healing after horizontal fracture: a case report with a 13-year followup. Dent Traumatol.

[4] Calişkan MK, Pehlivan Y (1996). Prognosis of root-fractured permanent incisors. Endod Dent Traumatol.

[5] Molina JR, Vann WF Jr, McIntyre JD, Trope M, Lee JY (2008). Root fractures in children and adolescents: diagnostic considerations. Dent Traumatol.

[6] Andreasen FM, Andreasen JO, Bayer T (1989). Prognosis of root-fractured permanent incisors--prediction of healing modalities. Endod Dent Traumatol.

[7] Feiglin B (1995). Clinical management of transverse root fractures. Dent Clin North Am.

[8] Linkow LI (1970). Theories and Technique of Oral Implantology. https://www.worldcat.org/title/theories-and-techniques-of-oral-implantology/oclc/114397.

[9] Ham KA, Witherspoon DE, Gutmann JL, Ravindranath S, Gait TC, Opperman LA (2005). Preliminary evaluation of BMP-2 expression and histological characteristics during apexification with calcium hydroxide and mineral trioxide aggregate. J Endod.

[10] Gurtu A, Singhal A, Mohan S (2012). Management of horizontal fracture. J Dent Sci.

